# Gambling Disorder in Male Violent Offenders in the Prison System: Psychiatric and Substance-Related Comorbidity

**DOI:** 10.1007/s10899-018-9785-8

**Published:** 2018-07-03

**Authors:** Carolina Widinghoff, Jonas Berge, Märta Wallinius, Eva Billstedt, Björn Hofvander, Anders Håkansson

**Affiliations:** 1 Faculty of Medicine, Department of Clinical Sciences Lund, Psychiatry, Lund University, Lund, Sweden; 2 Clinical Research Unit/Gambling Disorder Unit, Malmö Addiction Center, 205 02 Malmö, Region Skåne Sweden; 3 Faculty of Medicine, Department of Clinical Sciences, Lund, Child and Adolescent Psychiatry, Lund University, Lund, Sweden; 4 Regional Forensic Psychiatric Clinic, Växjö, Sweden; 5 Department of Psychiatry and Neurochemistry, Centre for Ethics, Law and Mental Health, Institute of Neuroscience and Physiology, The Sahlgrenska Academy at University of Gothenburg, Gothenburg, Sweden; 6 Gillberg Neuropsychiatry Centre, Institute of Neuroscience and Physiology, Sahlgrenska Academy at the University of Gothenburg, Gothenburg, Sweden; 7 Division of Forensic Psychiatry, Faculty of Medicine, Department of Clinical Sciences, Lund, Child and Adolescent Psychiatry, Lund University, Lund, Region Skåne Sweden

**Keywords:** Gambling disorder, Offenders, Substance use disorder, Psychiatric comorbidity

## Abstract

Gambling disorder is an addiction that can cause major suffering, and some populations seem to be more vulnerable than others. Offender populations have a remarkably high prevalence of gambling problems and they are also over-represented in a number of diagnoses related to gambling disorder, like substance use disorders and antisocial personality disorder. Yet, there are few studies investigating gambling disorder prevalence and related psychiatric comorbidity in this group. This study aims to investigate the prevalence of, and association between, gambling disorder and other psychiatric diagnoses in a sample of young, male violent offenders. Two hundred and sixty-four male offenders, all serving sentences for violent crimes (recruited between 2010 and 2012) participated in this study and went through comprehensive psychiatric evaluation, including assessment for Diagnostic and Statistical Manual of Mental Disorders 4th Edition criteria. Sixteen percent of the participants met criteria for gambling disorder. Antisocial personality disorder, cannabis, cocaine and anabolic steroids abuse were significantly more common among participants with gambling disorder. The gambling disorder group also showed significantly lower educational attainment. Cocaine abuse and failure to graduate elementary and middle school in expected time were independently associated with gambling disorder in a regression analysis. This study confirms the previously described high prevalence of gambling disorder in offenders. The psychiatric comorbidity was high and the problems had started early, with lower educational attainment in the gambling disorder group. The findings stress the importance of increased awareness of gambling problems among convicted offenders and of gambling research on young people with delinquent behavior. There is a need of more research to investigate this further, in order to develop preventive strategies and treatment.

## Background

Gambling disorder is known to cause severe harm on both individual and societal levels (Langham et al. 2016). Estimations of point prevalence of gambling disorder in adult populations vary between .2 and 2% globally (Lorains et al. 2011; Grant et al. 2016; Van Patten et al. 2017). While the prevalence estimations have been stable during the past decade, the proportion with the most serious problems seems to increase, and it has been shown that minority groups and people with low socioeconomic status are at higher risk of developing gambling problems than the general population (Abbott et al. 2014; Van Patten et al. 2017). These trends illustrate the need of preventive actions in vulnerable groups, and development of effective treatments.

According to a systematic review, the prevalence of gambling problems in offender populations is remarkably high, with an average of about 33%, and a high degree of variability (5–73%, problem and pathological gambling combined) in the estimates, depending on differences in assessment methods, size and quality of the studies (Williams et al. 2005). Despite this well known connection, and the fact that criminality is associated with a number of different psychiatric diagnoses, there are relatively few studies that focus on criminality and gambling. Both substance use disorders and antisocial personality disorder are highly overrepresented among offenders, and also connected to gambling disorder (Slutske et al. 2001; Pietrzak and Petry 2005; Lorains et al. 2011). During the past four decades, a total of 37 studies focusing on prevalence of gambling disorder in offender populations have been performed; 27 articles with varying methods are included in the review by Williams and colleagues (2005), five more are summed in the literature part of a study by Zurhold et al. (2014), and another five have been published the last years (Kerber et al. 2012; Turner et al. 2013; May-Chahal et al. 2016; Tessenyi and Kovacs 2016; April and Weinstock 2017). Only two of the mentioned studies include diagnostics based on the Diagnostic and Statistical Manual of Mental Disorders 4th Edition (DSM-IV) (American Psychiatric Association 2000) criteria, in both cases in form of a self-report scale (Turner et al. 2009, 2013). The South Oaks Gambling Screen is the most commonly used screening tool, but it is not sufficient for actual diagnoses, and has been criticized in various ways (Lesieur and Blume 1987; Volberg 2004). As far as the authors of this study know, there is no previous study on offenders performing full DSM diagnostics, including gambling disorder.

To summarize, previous studies indicate that gambling disorder is overrepresented among offenders, and that an increased knowledge about these complex connections is necessary for optimizing screening, possible treatment, and rehabilitation (Williams et al. 2005). There is, however, a pronounced lack of gambling disorder research based on diagnostics through full DSM assessments (Zurhold et al. 2014).

The primary aims of this study were to investigate the prevalence of, and association between, gambling disorder and other psychiatric diagnoses in a group of Swedish young, male violent offenders. Secondary aims were to compare the gambling and the non-gambling disorder groups concerning types of crimes and sociodemographic data.

## Methods

### Participants

The participants in this study originally took part in the Swedish research project DAABS (*the Development of Aggressive Antisocial Behavior Study*), which investigated a nationally representative cohort of violent offenders concerning mental and neurodevelopmental disorders (Wallinius et al. 2016). They were all men aged 18–25 years and recruited while serving a sentence for violent (including hands-on sexual) crimes in the Western region of the Swedish Prison and Probation Service during the period March 2010–July 2012. Nine different prisons were involved, ranging from high to low security facilities, and all violent offenders in the age group (corresponding to a fifth of the national population) were asked to participate. Since there was only one women’s prison in the defined area, no women were included due to power issues. Exclusion criteria were insufficient language skills (defined as the need of an interpreter for full participation) and short duration of stay at the current prison (≤ 4 weeks). Anonymous, basic information about the offenders who were excluded, or chose not to participate, was obtained and compared to the rest of the offenders to assess the representativeness of the sample.

Out of 421 imprisoned men of the right age and crime category, 23 were excluded due to insufficient language skills and 19 because of too short duration of prison stay. Of the remaining 379 clients, 109 declined participation leaving a total response rate of 71% (n = 270) among all of those who met the inclusion criteria. In six cases, there was not sufficient information from the clinical assessments to make a diagnostic evaluation about the presence of a gambling disorder, which yielded a group of 264 participants for this study.

In the group of men who declined participation (n = 109) there were no significant differences in mean age or type of index crime (general violent or sexual violent), compared to those who participated. However, in the smaller group that was excluded due to insufficient language skills, sexual violent crimes were overrepresented; 52% (n = 12) in comparison to 11% (n = 28) among the participants. In summary, the cohort of 264 men was considered representative for young Swedish male offenders convicted of these crimes.

## Procedure

### General Procedure

The eligible offenders received oral and written information about the study and were asked for informed consent. A small monetary compensation (200 SEK appr. USD25) was provided.

A preset protocol was followed, consisting of self-rating questionnaires, semi-structured diagnostic interviews and neuropsychological assessments. The questionnaires were completed by the participants before a full day of clinical assessments. The assessors were licensed psychologists with clinical experience from the field and specialized in the instruments used. All clinical data available from the Swedish Prison and Probation Service (i.e. prison medical records, detailed reports on previous living circumstances and history, and incidents during ongoing sanction) were read by the assessors and evaluated for quality.

### Psychosocial and Criminal Background

Information on psychosocial background, e.g. marital status, ethnicity, family background, schooling and former contact with the healthcare system, were collected by the assessor from file information and structured interviews. The criminal backgrounds of the participants were also mapped out through information from files and structured interviews.

### Diagnostic Assessments

Psychiatric assessments were based on the Structured Clinical Interview for DSM-IV Axis I Disorders (SCID-I) (First 1997), lifetime clinical disorders, and the SCID-II (First et al. 1997) for personality disorders. In order to assess the disorders not covered in the SCID (e.g. neurodevelopmental disorders and impulse control disorders, including gambling disorder/pathological gambling), an amendment, including a life-time DSM-IV symptom checklist of individual criteria or symptom definitions according to DSM-IV (American Psychiatric Association 2000), was added to the structured interview protocol. Final diagnoses were based on all available information, provided by the clinical interview, medical and other records, self-rating questionnaires, neuropsychological assessments, and the clinical impression of the respondent during the 5–6 h assessment, and assigned in consensus between the clinical psychologist and a senior psychologist and researcher (EB or BH), according to the LEAD-principle (Spitzer 1983), i.e. clinicians making decisions in consensus using all data available. Concerning the gambling disorder diagnoses, there was not much additional information available, and the definitive diagnoses were based on the structured DSM-IV symptom checklist (Billstedt et al. 2017; Hofvander et al. 2017).

Intellectual functioning was measured with the Wechsler Adult Intelligence Scales—third edition, (WAIS-III) (Wechsler 2002). In this study, the General Ability Index (GAI) (Tulsky et al. 2001) from WAIS-III was calculated. The Asperger Syndrome/high functioning autism Diagnostic Interview (ASDI) (Gillberg et al. 2001) was used to assess signs of an autism spectrum disorder (ASD). The ASDI is a combined interview and observation schedule for clinical assessments. For participants potentially meeting diagnostic criteria for an ASD disorder, when possible, an in-depth autism spectrum examination was carried out, including either a “Diagnostic Interview for Social and Communication disorders” (DISCO) (Wing et al. 2002), with parents/caregivers or an “Autism Diagnostic Observation Schedule” (ADOS) (Lord et al. 2000), with the participant. For a thorough description of the diagnostic procedures see Billstedt et al. (2017) and Hofvander et al. (2017).

### Substance Abuse

The diagnostics in this study were performed in the middle of the transition from DSM-IV to DSM-5, where the two concepts “substance abuse” and “substance dependence” were merged into “substance use disorder” with three degrees of severity (American Psychiatric Association 2000, 2013). Due to the extensive social problems and unconventional life situations of the participants, it was hard to assess the substance dependence criteria. Therefore, the PIs decided to use a limited set of criteria and in this study we report whether an individual fulfilled at least substance abuse diagnosis, based on the four DSM-IV substance abuse criteria, while a subject’s substance abuse diagnosis may or may not have progressed to a dependence diagnosis for that specific substance.

### Types of Crimes

Information about types of crimes was collected from files and structured interviews (Wallinius et al. 2016) and is reported as variables summarizing all reported (official or during interviews) crimes with a lifetime prevalence. Being convicted of a violent (including hands-on sexual) crime was an inclusion criteria for this study. All offenses were arranged in six groups: violent offences (i.e. murder/manslaughter, assault, unlawful threat, robbery, sexual offenses, and fire setting/arson), sexual offences, drug-related offences, property offences (i.e. theft, breaking and entering, and vandalism), traffic violations (i.e. driving under the influence, and driving without a license) and fraud. All categories include attempts as well as aggravated versions of the specific crimes listed above.

## Statistical Analysis

All data were anonymized, coded and gathered in a database. The calculations were performed using IBM SPSS Statistics for Mac, version 23.0 (IBM, Armonk, NY). A *p* value < .05 was considered statistically significant. All *p* values were two-tailed.

Initially, bivariate analyses were performed. Comparisons between groups (age) were assessed using the Student’s *t* test. Dichotomous variables are presented as absolute and relative frequency and comparisons between groups were calculated with Fisher’s Exact test. In order to restrain the false discovery rate among the 27 bivariate analyses of potential predictors in the multivariate model to .05 (i.e. the chance of any type I error among all of the 27 analyses) the Benjamini Hochberg (BH) correction procedure was performed on the *p* values from the analyses (Hochberg and Benjamini 1990). The rate of missing data was low, ranging from 0 to 1.5%.

Secondly, we used logistic regression analysis to investigate the significant variables associated with gambling disorder. The limited number of participants with gambling disorder (n = 43) makes the risk of overfitting a potential issue given the large number of possible variables to include in the multivariate regression model. A general recommendation to minimize the risk of overfitting in logistic regression analysis is a maximum of one independent variable for every 8–10 cases of the dependent variable (Vittinghoff and McCulloch 2007). We thus decided to limit the number of independent variables in the regression model to five, and we included the five variables with the lowest BH-corrected *p* values in the bivariate analyses. Missing data were handled by listwise deletion.

## Ethical Considerations

Informed written consent was provided by all the offenders before participation. They were given the opportunity to receive feedback on the preliminary results of the assessment, together with the opportunity of referral to the prison psychiatrist for further assessment and possible treatment. The monetary reward was considered low enough not to influence the free consent. The study was approved by the Research Ethics Committee at Lund University (Register No. 2009/405).

## Results

### Gambling Disorder

The prevalence of gambling disorder in the cohort was 16% (n = 43). Under the assumption that this cohort is representative for young Swedish male violent offenders, this corresponds to a 95% confidence interval for the true proportion of gambling disorder in this population at 12.0–21.3%.

The percentages of participants with and without gambling disorder reporting each of the pathologic gambling criteria are shown in Table 1.

**Table 1:** Frequency of diagnostic criteria for lifetime gambling disorder

Diagnostic criterion	Proportion positive in gambling disorder group (%)
Preoccupation with gambling	81.4
Needs to gamble with increasing amounts of money	79.1
Repeated unsuccessful efforts to control gambling	58.1
Withdrawal symptoms	69.8
Gambles as a way of escaping	53.5
Chasing losses	81.4
Lies to conceal the extent of involvement in gambling	74.4
Committed illegal acts to finance gambling	51.2
Jeopardized relationships, job etc.	23.3
Relies on others to provide money	14.0

### Sociodemographics

Sociodemographic variables are shown in Table 2. There were no significant differences between the gambling disorder and the non-gambling disorder group concerning age, marital status, country of birth (Sweden or other), or employment. When it comes to elementary and middle school graduation, however, significantly fewer in the gambling disorder group had graduated in expected time (BH-adjusted *p* value, *p*_BH_ = .027).

**Table 2:** Sociodemographic data by occurence of gambling disorder, lifetime

	Total sample	Gambling disorder group	Non gambling disorder group	*p* value*	BH-adjusted *p* value**
Age, mean (years)	22.3	22.6	22.3	.281	.380
Married/living together (%)	24.2	32.6	22.6	.175	.254
Born in Sweden (%)	73.5	72.1	73.8	.851	.884
Not graduated elementary and middle school in expected age	25.5	44.2	21.8	**.004**	**.027**
Unemployed before arrest	60.8	65.1	60.0	.610	.659

Associations that remain significant after BH-adjustment are presented in bold text

*Fisher’s exact test used for all categorical variables, Student’s *t* test used for all numerical variables

**Benjamini–Hochberg adjusted *p* values using all 27 *p* values displayed in Tables 2 and 3

### Substance Abuse

A total of 84.5% (n = 223) had at least one substance abuse diagnosis. There were significant differences between the groups considering cannabis abuse (*p*_BH_ = .043), cocaine abuse (*p*_BH_ < .001) and anabolic steroids abuse (*p*_BH_ = .027), where the specific substance abuse diagnoses were more common in the gambling disorder group.

### Other Psychiatric Diagnoses

All psychiatric diagnoses, their percentages and *p* values from the bivariate analysis are shown in Table 3.

**Table 3:** Psychiatric and substance abuse comorbidity by occurence of gambling disorder, lifetime

	Total sample, % (n)	Gambling disorder group, % (n)	Non gambling disorder group, % (n)	*p* value*	BH-adjusted *p* value**
Mental retardation	1.9 (5)	7.0 (3)	0.9 (2)	.032	.096
ADHD	43.5 (114)	53.5 (23)	41.6 (91)	.179	.254
Autism spectrum disorders	9.5 (25)	0.0 (0)	11.3 (25)	.019	.086
Conduct disorder	79.2 (209)	88.4 (38)	77.4 (171)	.149	.237
Substance abuse (any)	84.5 (223)	93.0 (40)	82.8 (183)	.109	.226
Alcohol	48.5 (128)	53.5 (23)	47.5 (105)	.508	.596
Sedatives	48.9 (128)	65.1 (28)	45.7 (100)	.029	.096
Cannabis	77.6 (204)	93.0 (40)	74.5 (164)	**.008**	**.043**
Central stimulants	48.9 (128)	60.5 (26)	46.6 (102)	.133	.226
Cocaine	40.8 (107)	74.4 (32)	34.2 (75)	**< .001**	**< .001**
Hallucinogens	34.0 (89)	48.8 (21)	31.1 (68)	.024	.093
Anabolic steroids	14.9 (39)	30.2 (13)	11.9 (26)	**.004**	**.027**
Inhalants	20.3 (53)	16.3 (7)	21.1 (46)	.540	.608
GHB	19.3 (51)	30.2 (13)	17.2 (38)	.058	.157
Heroin	33.8 (89)	39.5 (17)	32.7 (72)	.385	.495
Opioid analgesics	41.7 (110)	53.5 (23)	39.4 (87)	.093	.209
Methadone, buprenorphine	14.0 (37)	9.3 (4)	14.9 (33)	.472	.580
Psychotic disorders	7.6 (20)	7.0 (3)	7.7 (17)	1.00	1.00
Affective disorders	54.2 (143)	65.1 (28)	52.0 (115)	.134	.226
Anxiety disorders	51.7 (136)	62.8 (27)	49.5 (109)	.134	.226
Eating disorders	1.1 (3)	4.7 (2)	0.5 (1)	.070	.172
Antisocial personality disorder	64.0 (169)	83.7 (36)	60.2 (133)	**.003**	**.027**

Associations that remain significant after BH-adjustment are presented in bold text

*Fisher’s exact test used for all categorical variables

**Benjamini–Hochberg adjusted *p* values using all 27 *p* values displayed in Tables 2 and 3

The diagnosis of mental retardation was significantly more common in the gambling disorder group according to the initial analysis (*p* = .032) but after correction with the BH method, the *p* value was no longer significant due to the small sample size (*p*_BH_ = .096).

Antisocial personality disorder was significantly more common in the gambling disorder group in the bivariate analysis (*p*_BH_ = .027).

### Regression Analysis

Five variables showed significant differences between the gambling disorder and non-gambling disorder groups in the bivariate analyses (see above and Tables 2, 3); elementary and middle school graduation in expected time, cannabis abuse, cocaine abuse, anabolic steroids abuse and antisocial personality disorder and were chosen for logistic regression analysis (see Table 4). In this analysis, cannabis abuse, anabolic steroids abuse and antisocial personality disorder were not independently associated with gambling disorder. Gambling disorder was significantly more common among those who had not graduated elementary and middle school in expected time (AOR 2.79, CI 1.33–5.87, *p* = .007) and among those who had cocaine abuse (AOR 4.11, 1.75–9.63, *p* = .001). Nagelkerke R Square of the model was .219, χ^2^ = 36.2, with *p* < .001.

**Table 4:** Logicistic regression on occurence of gambling disorder

	OR (95% CI)	AOR (95% CI)	*p* value*
Not graduated elementary and middle school in expected age	2.85 (1.44–5.64)	2.79 (1.33–5.87)	**.007**
Cannabis abuse	4.55 (1.36–15.3)	1.45 (0.35–6.05)	.607
Cocaine abuse	5.59 (2.67–11.7)	4.11 (1.75–9.63)	**.001**
Anabolic steroids abuse	3.22 (1.49–6.94)	1.47 (0.62–3.46)	.381
Antisocial personality disorder	3.40 (1.45–7.99)	1.88 (0.73–4.87)	.191

Associations that remain significant in the multivariable regression model are presented in bold text

*p values from the adjusted logistic regression analysis

### Types of Crimes

Types of crimes by occurrence of gambling disorder are shown in Table 5. Initially there were significant associations between gambling disorder and drug-related (*p* = .022) and traffic violations (*p* = .036) but after a BH-correction for these five analyses, the associations were no longer significant (*p*_BH_ = .090 and *p*_BH_ = .090).

**Table 5:** Types of crimes by occurence of gambling disorder, lifetime

	Total sample, % (n)	Gambling disorder group, % (n)	Non gambling disorder group, % (n)	*p* value*	BH-adjusted *p* value**
Violent offenses	100 (264)	100 (43)	100 (231)	N/A	
Sexual offenses	11.8 (31)	11.6 (5)	11.8 (26)	1.00	1.00
Drug-related offenses	74.0 (194)	88.4 (38)	71.2 (156)	.022	.090
Property offenses	87.9 (232)	90.7 (39)	87.3 (193)	.798	.998
Traffic violations	64.6 (170)	79.1 (34)	68.1 (136)	.036	.090
Fraud	25.9 (68)	30.2 (13)	25.0 (55)	.454	.757

*Fisher’s exact test used for all categorical variables

**Benjamini–Hochberg adjusted *p* values using the 5 *p* values displayed in this table

## Discussion

### Prevalence and the Need of Attention

The main finding of this study is the high prevalence of gambling disorder in violent offenders, comprehensively assessed with DSM-IV-based interviews, confirming the results from previous research (Williams et al. 2005). This prevalence is about 40 times higher than in the general Swedish population. The large epidemiological study SWELOGS (Swedish Longitudinal Gambling Study) has shown that general gambling in Sweden appears to have reached a plateau (Romild et al. 2014), but the proportion of problem gamblers in the population remains the same and gamblers with the most serious problems are even increasing.

The pronounced relationship between criminality and gambling disorder could form an incentive to pay more attention to gambling problems in the correctional system. Gambling has been reported as a usual, perhaps even normative, part of prison life (McEvoy and Spirgen 2012) which likely impairs the chances of rehabilitation, and gambling severity is a significant predictor of criminal recidivism according to a study by April and colleagues (Lahn 2005; April and Weinstock 2017). The causal relations between gambling disorder and criminality are not fully understood (Turner et al. 2013; May-Chahal et al. 2016), but there is an obvious risk for criminal relapse when a person is in gambling debt, and this two-way connection motivates increased awareness and availability of gambling disorder treatment to people with a criminal lifestyle.

Concerning types of crimes, there were no significant differences between the gambling disorder and the non-gambling disorder group. However, this study—including only violent offenders—does confirm the picture that gambling disorder might be common among offenders regardless of type of crime, and not mainly connected with economic crime or property crime, which has also been suggested (Turner et al. 2009, 2013; Cuadrado and Lieberman 2012).

The frequency of each DSM gambling criterion in the gambling disorder group in this study is interesting and shows both similarities and differences compared to previous research. About 51% of the gambling disordered group reported having committed a criminal act to finance their gambling, which is similar to reports from Abbott and colleagues, Lahn and colleagues and the Williams’ review (Abbott et al. 2005; Lahn 2005; Williams et al. 2005). However, when compared to other gambling disordered groups, the offenders with gambling disorder in this study to a markedly less extent reported having jeopardized or lost a significant relationship, job etc. (23.3%) or relied financially on others because of gambling (14.0%) (Granero et al. 2014; Christensen et al. 2015). A possible explanation could be that this group of men is limited in their ability to perceive and assess consequences of their actions. Previous research has shown impaired risk assessment in offenders, which probably is connected to the high prevalence of antisocial personality disorder (Pachur et al. 2010; May-Chahal et al. 2016). Perhaps their relatively young age and complicated life situations could affect what kind of relationships they are in, and their appreciation of them. Pachur and colleagues suggest that enhanced thinking skills programs for offenders, aimed at reducing recidivism through changing attitudes, could be more successful with an increased focus on risk taking (Pachur et al. 2010). Many criminal problem gamblers don’t see their gambling as problematic at all (Lahn 2005), which further emphasizes the need of cognitive behavioural interventions.

### Early Problems and Detection

We have also showed that, even if this group of convicted men has had a substantial amount of early onset problems with e.g. schooling and demonstrates a high prevalence of conduct disorder, the participants with gambling disorder stand out with significantly worse results concerning elementary and middle school graduation. This is interesting in the perspective of the development of early deviant behavior, and previous publications on the same population show parallel results; school adjustment problems (e.g. truancy, bullying and incomplete schooling) were the most distinct significant predictors of later aggressive behavior (measured as total score on Life History of Aggression) (Wallinius et al. 2016). In line with this, an early criminal career has been associated with higher loss chasing in gambling according to a study by May-Chahal et al. (2016).

Swedish epidemiological research has shown that gambling problems are highly overrepresented among young, marginalized men (Abbott et al. 2014) and it is probable that gambling problems and antisocial behavior develop simultaneously (Slutske et al. 2001) and perhaps catalyze each other. A part of the longitudinal study on the Dunedin birth cohort concludes that gambling disorder in young adults has much in common with addictive disorders and other externalizing behaviors, from a personality perspective (Slutske et al. 2005). Thus, young men with impulsive and delinquent behavior are clearly in the risk zone for gambling disorder and should be targets for preventive actions and treatment.

### Psychiatric Comorbidity

When it comes to the extremely high prevalence of psychiatric disorders in this cohort, it speaks for the need of competent psychiatric care for young offenders (Al-Rousan et al. 2017). The criminal gamblers may be predisposed to gambling problems and form an interesting group from a biological point of view. They could represent a certain “antisocial pathway” to gambling disorder; characterized by low impulse control, high aggression levels and multiple drug use (Blaszczynski and Nower 2002; Valleur et al. 2016; Allami et al. 2017). Gupta and colleagues suggest that the “pathways model” is applicable also for adolescents, confirmed by latent class analysis which showed a distinct impulsive and antisocial subgroup among the young problem gamblers (Gupta et al. 2013). The antisocial gamblers often start at a young age (Valleur et al. 2016; Allami et al. 2017) and gambling may play a role in the evolvement of an antisocial lifestyle (Slutske et al. 2001). According to various studies looking at gambling and personality, problem gambling could be considered part of a cluster of externalizing pathology. The personality profiles of pathological gamblers and substance abusers are often dominated by negative affect and unconscientious—and disagreeable disinhibition, a combination of traits also closely connected to antisociality and impulsivity (Maclaren et al. 2011). It is believed that it is of great importance to pay more attention to the associations between both gambling disorder and substance use disorders, and criminal maintenance (McEvoy and Spirgen 2012). There is an evident connection between criminality and gambling, and even though the chronology is complex, the research on this cohort enlightens the need of efforts early in life to affect the development of both social and psychiatric problems (Nilsson et al. 2016). Further observation of this group of gamblers, through screening and the offering of treatment, would be necessary to evaluate the possible benefit of treatment interventions.

In the present study, we found significantly higher occurrence of antisocial personality disorder and three substance abuse diagnoses in the gambling disorder group; cannabis, anabolic steroids and cocaine. The variables that were independently associated with gambling disorder in the regression model were elementary and middle school graduation and cocaine abuse. Possible causal relations behind these findings are not possible to determine through this study, but our results enlighten the need of further studies.

The comorbidity between gambling disorder and substance use disorders has previously been demonstrated, and more serious alcohol problems have been shown to correlate with more severe gambling disorder and higher general dysfunction (Lorains et al. 2011; Del Pino-Gutierrez et al. 2016). Pietrzak and colleagues also demonstrated illicit drug use and severity of gambling problems to be indicators of antisocial personality disorder in treatment seeking gamblers (Pietrzak and Petry 2005). The presence of substance abuse diagnoses was very high in this study, and enlightens the need of the offering of treatment to possibly decrease relapses in gambling disorder, and other addictive disorders, and criminality. There were no significant differences between groups considering alcohol abuse, but the prevalence is considerably high in the whole cohort; 48.5%, compared to approximately 4% in the general population (Andréasson 2011). The significant overrepresentation of cocaine abuse in the gambling disorder group, still significant in the regression analysis, is interesting and might speak for a pronounced reward seeking behavior, together with problematic decision-making. Dufour and colleagues found an overrepresentation of problem gamblers among community cocaine users (Dufour et al. 2016), and alterations in neural structure in specific parts of the orbitofrontal cortex—playing an important role for drive and responsibility—have been connected to both cocaine-use disorder and gambling disorder (Adinoff et al. 2003). The field is complex; so far the studies are small and somewhat ambiguous, but there are interesting findings indicating the need of further analyses of addictive behaviors in this context (Adinoff et al. 2003; Yip et al. 2017). Our results show a connection between cocaine and gambling disorder in the studied group, even though a lot still remains unclear when it comes to the explaining pathobiology.

### Limitations

The study is limited by the relatively small size of the cohort, which may have lead to undetected type II errors. The exclusion of 23 participants due to language problems may have affected the results, since men born outside Sweden seem to be at higher risk for gambling problems (Abbott et al. 2014). It cannot be precluded that the group of 109 men who declined participation in the study was different in any way considering prevalence of gambling disorder and other variables investigated. Analysis of the non-responders was limited to age and type of crime.

The cohort itself was defined by a number of factors that may limit the generalizability of the results because of selection biases. The age span was narrow (18–25 years), no females were included and all the participants were convicted of violent (including hands-on sexual) crimes. In addiction medicine research, maybe particularly concerning gambling disorder, it is important to underline that there are cross-cultural differences both within—and between countries. The current sample is a quite specific group in the Swedish population, which may of course limit the generalizability also to other similar populations (Medeiros et al. 2015; Raylu and Oei 2004).

Many parts of our data were selected retrospectively, hence a recurrent risk for recall bias. All diagnoses were based on professional assessments, and as always there are elements of subjectivity. The substance abuse diagnoses were comprehensively based on DSM-IV, but substance dependence criteria were not fully assessed. The gambling disorder diagnoses were based on a structured list of DSM-IV criteria, but no structured validated instrument was used, which may have affected the diagnostic validity of gambling disorder.

When performing multiple comparisons there is always a risk for type 1 errors. We handled this risk by using the BH method in which all *p* values from the bivariate analyses were adjusted to limit the total false discovery rate to 5%, prior to the regression analysis (Hochberg and Benjamini 1990).

A main drawback with the cross-sectional design is the lack of time perspective, which also rules out the possibility to estimate risks and possible causal relationships.

## Conclusions

In summary, this study investigated gambling disorder in a cohort of young men convicted for violent and/or sexual crimes and showed a high prevalence, 16%, when assessed with a structured DSM-based diagnostic instrument. The clinical implications could be divided in two sections; at first the direct need of illuminating gambling problems among offenders; both to provide treatment for the gambling problems themselves, but also as a conceivable way of diminishing the risk of criminal recidivism. Secondly, our findings emphasize the importance of early attention to gambling problems in vulnerable groups; here represented by young men with schooling problems and early-onset delinquent behavior.
