# Correction to: Gambling Disorder in Male Violent Offenders in the Prison System: Psychiatric and Substance‑Related Comorbidity

**DOI:** 10.1007/s10899-021-10102-6

**Published:** 2022-01-29

**Authors:** Carolina Widinghoff, Jonas Berge, Märta Wallinius, Eva Billstedt, Björn Hofvander, Anders Håkansson

**Affiliations:** 1 Department of Clinical Sciences Lund, Psychiatry, Faculty of Medicine, Lund University, Lund, Sweden; 2 Clinical Research Unit/Gambling Disorder Unit, Malmö Addiction Center, 205 02 Malmö, Region Skåne Sweden; 3 Faculty of Medicine, Department of Clinical Sciences, Lund, Child and Adolescent Psychiatry, Lund University, Lund, Sweden; 4 Regional Forensic Psychiatric Clinic, Växjö, Sweden; 5 Department of Psychiatry and Neurochemistry, Centre for Ethics, Law and Mental Health, Institute of Neuroscience and Physiology, The Sahlgrenska Academy at University of Gothenburg, Gothenburg, Sweden; 6 Gillberg Neuropsychiatry Centre, Institute of Neuroscience and Physiology, Sahlgrenska Academy at the University of Gothenburg, Gothenburg, Sweden; 7 Division of Forensic Psychiatry, Faculty of Medicine, Department of Clinical Sciences, Lund, Child and Adolescent Psychiatry, Lund University, Lund, Region Skåne Sweden

## Correction to: Journal of Gambling Studies (2019) 35:485–500 https://doi.org/10.1007/s10899-018-9785-8

The original version of this article unfortunately contains a mistake. The corrected details are given below.

- Under heading “Methods”: In second paragraph of sub-heading "Participants" the following sentence "In six cases, there was not sufficient information from the clinical assessments to make a diagnostic evaluation about the presence of a gambling disorder, which yielded a group of 264 participants for this study." should read as "In six cases, there was not sufficient information from the clinical assessments to make a diagnostic evaluation about the presence of a gambling disorder, which yielded a group of 263 participants for this study."
- The decimals in percentages and *p*-values in all five tables should be updated. The corrected Tables 1, 2, 3, 4, 5 are given below.

**Table 1:** Frequency of diagnostic criteria for gambling disorder, lifetime

Diagnostic criterion	Proportion positive in gambling disorder group (%)
Preoccupation with gambling	81.0
Needs to gamble with increasing amounts of money	81.0
Reported unsuccessful efforts to control gambling	57.1
Withdrawal symptoms	71.4
Gambles as a way of escaping	52.4
Chasing losses	81.0
Lies to conceal the extent of involvement in gambling	76.2
Committed illegal acts to finance gambling	52.4
Jeopardized relationships, job etc	23.8
Relies on others to provide money	14.3

**Table 2:** Sociodemographic data by occurrence of gambling disorder, lifetime

	Total sample	Gambling disorder group	Non gambling disorder group	*p*-value*	BH-adjusted *p*-value**
Age, mean (years)	22.3	22.6	22.3	0.283	0.364
Married/living together (%)	24.3	33.3	22.6	0.168	0.267
Born in Sweden (%)	73.4	71.4	73.8	0.849	0.882
Not graduated elementary and middle school in expected age	25.5	45.2	21.7	0.003	**0.027**
Unemployed before arrest	60.7	64.3	60.0	0.731	0.789

Associations that remained significant after BH-adjustment are presented in bold text

*Fisher’s exact test used for all categorical variables. Student’s t test is used for all numerical values

**Benjamini–Hochberg adjusted *p*-values using all 27 *p*-values displayed in Tables 2 and 3

**Table 3:** Psychiatric and substance abuse comorbidity by occurrence of gambling disorder, lifetime

	Total sample, % (n)	Gambling disorder group, % (n)	Non gambling disorder group, % (n)	*p*-value*	BH-adjusted *p*-value**
Mental retardation	1.90 (5)	7.10 (3)	0.90 (2)	0.031	0.105
ADHD	43.3 (113)	52.4 (22)	41.6 (91)	0.234	0.316
Autism spectrum disorders	9.50 (25)	0.0 (0)	11.3 (25)	0.019	0.086
Conduct disorder	79.1 (208)	88.1 (37)	77.4 (171)	0.148	0.266
Substance abuse (any)	84.4 (222)	92.9 (39)	82.8 (183)	0.110	0.248
Alcohol	48.3 (127)	52.4 (22)	47.5 (105)	0.615	0.692
Sedatives	48.7 (127)	64.3 (27)	45.7 (100)	0.029	0.105
Cannabis	77.5 (203)	92.9 (39)	74.5 (164)	0.008	**0.043**
Central stimulants	48.7 (127)	59.5 (25)	46.6 (102)	0.133	0.257
Cocaine	40.6 (106)	73.8 (31)	34.2 (75)	< 0.001	** < 0.001**
Hallucinogens	33.7 (88)	47.6 (20)	31.1 (68)	0.049	0.147
Anabolic steroids	14.9 (39)	31.0 (13)	11.9 (26)	0.003	**0.027**
Inhalants	20.0 (52)	14.3 (6)	21.1 (46)	0.401	0.471
GHB	19.0 (50)	28.6 (12)	17.2 (38)	0.090	0.221
Heroin	34.0 (89)	40.5 (17)	32.7 (72)	0.375	0.460
Opioid analgesics	41.4 (109)	52.4 (22)	39.4 (87)	0.127	0.257
Methadone, buprenorphine	13.7 (36)	7.10 (3)	14.9 (33)	0.226	0.316
Psychotic disorders	7.6 (20)	7.10 (3)	7.7 (17)	1.00	1.00
Affective disorders	54.0 (142)	64.3 (27)	52.0 (115)	0.177	0.267
Anxiety disorders	51.5 (135)	61.9 (26)	49.5 (109)	0.178	0.267
Eating disorders	1.10 (3)	4.80 (2)	0.50 (1)	0.067	0.181
Antisocial personality disorder	63.9 (168)	83.3 (35)	60.2 (133)	0.005	**0.034**

Associations that remained significant after BH-adjustment are presented in bold text

*Fisher’s exact test used for all categorical variables

**Benjamini–Hochberg adjusted *p*-values using all 27 *p*-values displayed in Tables 2 and 3

**Table 4:** Logistic regression on occurrence of gambling disorder

	OR (95% CI)	AOR (95% CI)	*p*-value*
Not graduated elementary and middle school graduation in expected time	2.98 (1.50–5.91)	2.89 (1.37–6.10)	**0.005**
Cannabis abuse	4.44 (1.32–14.93)	1.46 (0.35–6.09)	0.601
Cocaine abuse	5.41 (2.58–11.37)	3.93 (1.67–9.27)	**0.002**
Anabolic steroids abuse	3.33 (1.54–7.20)	1.54 (0.65–3.63)	0.329
Antisocial personality disorder	3.31 (1.41–7.78)	1.83 (0.71–4.75)	0.215

The model had a Nagelkerke R Square of 0.217, χ^2^ = 35.5, with *p* < 0.001

Associations that remained significant in the multivariable regression model are presented in bold text

*p-values from adjusted logistic regression analysis

**Table 5:** Types of crimes by occurrence of gambling disorder

	Total sample, % (n)	Gambling disorder group, % (n)	Non gambling disorder group, % (n)	*p*-value*	BH-adjusted *p*-value**
Violent offenses	100 (263)	100 (42)	100 (221)	N/A	
Sexual offenses	11.8 (31)	11.9 (5)	11.8 (26)	1.00	1.00
Drug-related offenses	73.9 (193)	88.1 (37)	71.2 (156)	0.022	0.055
Property offenses	87.8 (231)	90.5 (38)	87.3 (193)	0.797	0.996
Traffic violations	64.9 (170)	81.0 (34)	68.1 (136)	0.021	0.055
Fraud	26.0 (68)	31.0 (13)	25.0 (55)	0.444	0.740

*Fisher’s exact test used for all categorical variables

**Benjamini–Hochberg adjusted *p*-values using the five *p*-values displayed in this table

